# Accelerated Radiotherapy with Concurrent Chemotherapy in Locally Advanced Head and Neck Cancers: Evaluation of Response and Compliance

**DOI:** 10.31557/APJCP.2020.21.5.1399

**Published:** 2020-05

**Authors:** Karim Mashhour, Hisham Atef, Ahmed Selim, Mostafa A Moez, Hussam Zawam, Yasser Abo-Madyan

**Affiliations:** 1 *Kasralainy Center of Clinical Oncology and Nuclear Medicine, Cairo University, Cairo, Egypt. *; 2 *Physics department at Kasralainy Center of Clinical Oncology and Nuclear Medicine, Cairo University, Cairo, Egypt. *; 3 *Department of Radiation Oncology, University Medical Center Mannheim, University of Heidelberg, Mannheim, Germany. *

**Keywords:** IMRT, SIB, toxicity compliance

## Abstract

**Purpose:**

Concurrent chemo-radiotherapy (CCRT) is the primary treatment modality for locally advanced head and neck squamous cell cancer patients (LAHNSCC). Intensity modulated radiotherapy (IMRT) with simultaneous integrated boost (SIB) and concurrent chemotherapy is not broadly implicated in our region mainly because of the lack of experience. This study aims at evaluating the response and compliance of this approach in our patients.

**Methods:**

Forty patients with LAHNSCC were included and 50% received induction chemotherapy. All the patients were treated with IMRT-SIB radiotherapy for 70Gy over 33 daily fractions. Weekly cisplatin (40mg/m^2^) was administered during the radiation course.

**Results:**

With median follow-up of 1.5 years, LC was achieved in 82.5% of cases and distant control rate was 90%. More than 5 interrupted radiation sessions and GTV volume > 50 cc significantly affected LRC (P= 0.02 and 0.001 respectively). Eighty percent of cases experienced grade 3 or 4 toxicities. Induction chemotherapy and PTV-70 volume >150 cc significantly affected the degree of toxicities (P=0.018 and 0.0001 respectively).The 2 years disease free survival (DFS) was 77%. ECOG PS, large GTV volume (> 50 cc) and RT interruption (>5 sessions) had negative impact on DFS (P= 0.041, 0.002 and 0.001 respectively). The 2 years overall survival (OS) was 87%. Radiation interruption (> 5 sessions) was the only factor which had significant detrimental effect on OS (P= 0.001).

**Conclusion:**

Induction chemotherapy seems to have a negative impact on patient’s compliance to CCRT. Bulky tumors and prolonged radiation interruptions were associated with significantly lower LRC, DFS and OS.

## Introduction

Head and neck squamous cell carcinoma (HNSCC) ranks among the most common cancers worldwide (Ferlay et al., 2015), with estimated about 63,000 new cases and 13,000 deaths in the USA only in 2017 (Siegel et al., 2017). In Europe, it represents about 4% of all cancers with 140,000 new cases in 2012 (Gatta et al., 2015). According to the Middle East Cancer Consortium, Egypt has one of the highest incidence rates of HNSCC in the region (Freedman et al., 2006). 

In the past few decades, the use of concurrent chemo-radiotherapy as the primary treatment modality for locally advanced head and neck cancer patients has been growing rapidly (Bourhis et al., 2012; Pignon et al., 2009).The benefits of adding concurrent chemotherapy to the standard RT has been validated in the meta-analysis of chemotherapy on head and neck cancer (MACH-NC), which confirmed an absolute 5-year survival benefit when adding chemotherapy of 4.5% (HR 0.88, p < 0.0001) (Pignon et al., 2009). Another meta-analysis showed that modified fractionation in addition to concurrent chemotherapy is more efficacious than modified fractionation alone (Budach et al., 2006). In the Radiation Therapy Oncology Group (RTOG) 0129 study; patients who received only 1 cycle of cisplatin had inferior outcomes compared to those who received the total prescribed dose (Ang et al., 2010). 

Dose fractionation and overall treatment time can have different implications on tumor control and toxicity (Teo et al., 2006).Alteration of the fractionation schedules with escalation of the dose delivered has been shown to improve overall survival (Budach et al., 2006), on the cost of increasing acute side effects (Bensadoun et al., 2006).With the implementation of intensity modulated radiotherapy (IMRT) to deliver simultaneous integrated boost (SIB), it could be possible to reduce these side effects while retaining the survival benefit (Clavel et al., 2012).

However the technique of IMRT with SIB and concurrent chemotherapy is not broadly applied in our region, partly because of the lack of experience with this technique and partly because of the lack of regional studies that describe patients’ response and tolerance.

*Aim of work*

The aim of this prospective study is to evaluate the response and toxicity of concurrent chemoradiotherapy using IMRT with SIB in locally advanced head and neck squamous cell cancer (LA-HNSCC) patients and to determine factors that lead to interruption of the radiation therapy sessions or omission of the planned chemotherapy.

## Materials and Methods

*Patients and methods*

*Study population*

Forty patients with LA-HNSCC were included. Recruitment started at the beginning of July 2016 and ended in November 2018. An acceptance from our institutional ethical and scientific committees was taken on the study design. A written consent was taken from all patients before their recruitment in our study. This study was approved by the ethics and clinical trials panel of the Cairo University Center of Clinical Oncology for conducting this non-funded thesis. On behalf of all authors, the corresponding author states that there is no conflict of interest.

*Selection criteria*

Included were patients with locally advanced head and neck cancers (stage 3 and 4), with pathologically confirmed squamous or undifferentiated carcinomas, age range from 18 to 70 years, ECOG performance status (PS) from 0-2 and creatinine clearance > 60 ml/min. Patients with metastatic disease or with history of another malignant disease were excluded.

*Details of therapy*

Patients underwent a pre-treatment evaluation, including a complete medical history and physical examination, computed tomography (CT) and/or MRI of head and neck region, direct flexible fibro optic endoscopic examination, chest X-ray or thoracic CT. Associated comorbidities were assessed using the Adult Comorbidity Evaluation-27 (ACE-27) (Bang et al., 2000).

Patients were set up in supine position and immobilized on a head support pad using a customized head-and-shoulder shell (S- type, Aquaplast, USA). All patients were scanned from skull vertex to mid-chest, with 2.5 mm slice thickness. Intravenous contrast was used in order to help in the definition of cervical nodes. CT images were then transferred to the Eclipse TPS (v8.6) via “DICOM” network. 

Cisplatin at a dose of 40mg/m2 was administered on weekly basis during the treatment course to a maximum of seven cycles.

The gross tumor volume (GTV) was defined as the macroscopic disease including all positive lymph nodes detected by clinical examination and radiological imaging. 

The CTVgross disease was composed of GTV with a 10-mm margin. Near the neural structures, the margin was reduced to as little as 1 mm. The CTVsubclinical disease was composed of CTV gross disease in addition to other areas at high risk of microscopic spread. Another CTV [CTVlow-risk subclinical disease] was delineated as the low level neck nodes (levels 4 and 5b) in certain cases with node negative disease. The delineation of cervical lymph node stations was based on the published consensus guidelines (Gregoire et al., 2003).

The PTVs were generally a 5-mm expansion of each of the CTVs to account for potential setup errors and patient motion. Similarly, the margin around the CTV was limited to 1 mm near the neural structures.

Patients were planned for inverse IMRT with the modality of step and shoot using Eclipse Planning System (version 8.6, from Varian Medical Systems).

Analog to the RTOG 0225 study (Lee et al., 2009), the dose to the PTVgross disease was prescribed as 69.96 Gy in 2.12 Gy per fraction, the dose to the PTV subclinical disease was 59.4 Gy in 1.8 Gy per fractions, and the dose to the PTVlow-risk subclinical disease was 54.12 Gy in 1.64 Gy per fractions. The prescribed doses were delivered in 33 once daily fractions, five fractions per week.

*Toxicity evaluation*

The Common Toxicity Criteria for Adverse Events [CTCAE v. 4.03](CTCAE 2010) was used for toxicity evaluation. 

Acute toxicities were recorded on a weekly basis till three months post-treatment while the late toxicities were documented thereafter until the latest follow up visit of the patient.

*Statistical analysis*

Statistics were done using SPSS software (Statistical package for social science) v.19. Descriptive statistics were presented as number and percentage (frequency distribution). 

On follow up, loco-regional control (LRC) was defined as negative appearance of any visible tumoral re-growth in the primary area and/or draining lymphatics while distant control (DC) was defined as no evidence of distant metastasis. Disease free survival (DFS) was defined as no evidence of disease whether local or systemic from the date of diagnosis to the date of documented death or loss of follow up. Overall survival (OS) was defined from the date of diagnosis to the date of documented death or loss of follow up.

Kaplan-Meier analysis with a log-rank test was used for survival analysis. Cox regression models were used to estimate hazard ratios and 95% conﬁdence intervals (CIs) for the risk of death. Factors were considered significant when p is < 0.05.

## Results

*Patients profiling*

Analysis of the data showed that most of our patients were males (65%). Median age was 49 years (range 19-69). ECOG PS at presentation was 0-1 in 95% of cases. Adult comorbidity evaluation (ACE – 27) grade was 0-1 in 92.5% of cases, only 3 patients had ACE grade 2 at presentation. Nasopharyngeal carcinoma represented the most common head and neck primary cancer (60% of cases), followed by oropharyngeal cancer (17.5%). Other characteristics are presented in Table 1. 

*Treatment interruptions*

Twenty patients (50% of cases) received induction chemotherapy (in the form of docetaxel, cisplatin and 5-flurouracil - TPF) before definitive chemoradiotherapy. Generally induction chemotherapy was used in case of bulky disease or to avoid delay of treatment initiation in case of long waiting list on the teletherapy machine.

In the whole group of patients, radiation interruption was documented in 19 patients (47.5% of cases). The main causes of interruption were unplanned machine breakdown (31%), development of treatment side effects (28%), preplanned holidays (23%) and patient related social issues (18%). The gaps were managed by adding weekend sessions in 31.5%, extra sessions in 26.5% and by both of them in 15.5% of the cases. In the remaining 26.5% of cases, the gaps were not compensated. The total number of interrupted RT days was 113 days.

Median radiation time for the whole group was 50 days (range 45 – 69 days). In patients who had RT interruption, the median treatment time was 56 days (range 51 – 69 days) and the median number of interrupted sessions was 6 sessions (range 3 – 14 sessions). 

The planned total dose of cisplatin during radiation was 280 mg/m^2^(40 mg/m^2^ weekly for 7 weeks). Only 13 patients (32.5%) completed the planned chemotherapy dose, while most of the patients (57.5% of cases) have omitted 1-2 chemotherapy cycles due to either neutropenia (45% of cases) or mucositis (17.5% of cases). 

*Disease control*

The median follow up was 18 months (range 14-33 months). Locoregional control (LRC) was achieved in 33 out of 40 patients (82.5% of cases). Four patients developed distant failure (2ry bone and lung metastases) leading to distant control rate of 90%. Analysis of the factors that affect LRC are shown in Table 2. More than 5 interrupted RT sessions and large GTV volume (> 50 cm3) were the only statistically significant factors while not receiving all planned concurrent chemotherapy cycles was of borderline significance. 

*Toxicity*

*Early hematological toxicities*

Eleven patients (27.5 %) developed grade 3 neutropenia. No other grade 3 or 4 hematological toxicities were noticed.

*Early Non hematological toxicities*

High grade mucositis (grade 3 or 4) was noticed in 75% of cases. Four patients (10%) developed grade 4 mucositis. Those patients were hospitalized and RT sessions were interrupted. They were treated from dehydration, neutropenia, fever and oropharyngeal candidiasis. Feeding gastrostomy was inserted in 2 patients. After being successfully managed, they completed their course of treatment.

Other high grade toxicities include; grade 3-4 xerostomia in 14 patients (35% of cases), grade 3 oral pain in 25 patients (62.5%), grade 3 laryngitis in 13 patients (32.5%), grade 3 dysphagia in 19 patients (47.5%), and grade 3 radiation dermatitis in 6 patients (15%).

*Late toxicities (after 90 days from the end of RT sessions)*

Grade 2 xerostomia was sustained in 40% of cases and grade 2 dysgeusia in 70% of cases. Only 2 patients (5%) developed grade 3 xerostomia and dysphagia. Both of them were hospitalized and 1 patient needed nasogastric tube insertion for proper alimentation. No grade 4 toxicities were noticed. 

*Factors affecting development of high grade toxicities*

Thirty two patients (representing 80%) have experienced grade 3 or 4 toxicities, with mucositis representing the most common toxicity occurring in 32 patients (75% of cases). Factors that might have an impact on the development of severe toxicities were analyzed in table 4. Induction chemotherapy [p = 0.018] and large GTV volume (> 50cm3) [p = 0.013] were the only statistically significant factors.

*Effect of induction chemotherapy on high grade toxicities and treatment delay*

**Figure 1:**
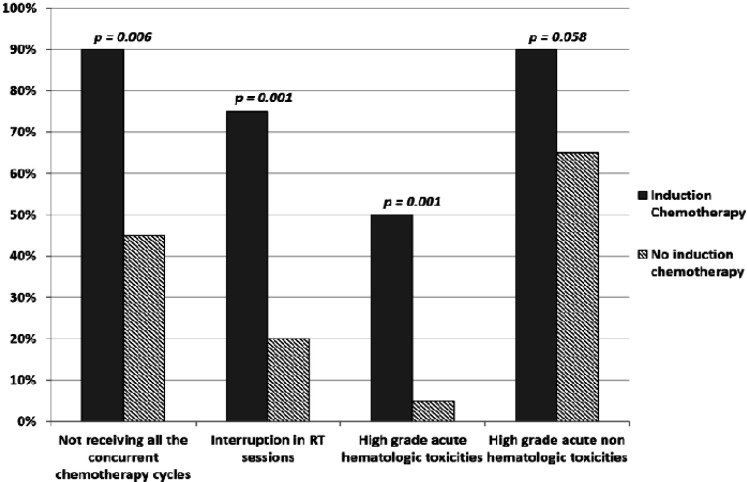
Bar Chart Showing the Relation between Induction Chemotherapy with Treatment Delay and Development of High Grade Toxicities and Its Statistical Significance (Calculated by 2 Sided Pearson Chi-Square Test)

As seen in Figure 1 patients who received induction chemotherapy suffered from significantly more high grade toxicities, both hematological and non-hematological, (p = 0.001 and 0.058 respectively). Ninety percent of patients who received induction chemotherapy did not complete the full dose of the concurrent cisplatin as opposed to 45% of those who didn’t receive induction therapy [p = 0.006]. Furthermore, they had more interruptions in their RT sessions (75% in the induction group vs 20% in the non-induction group, p = 0.001).

*Factors affecting disease free survival*

**Figure 2:**
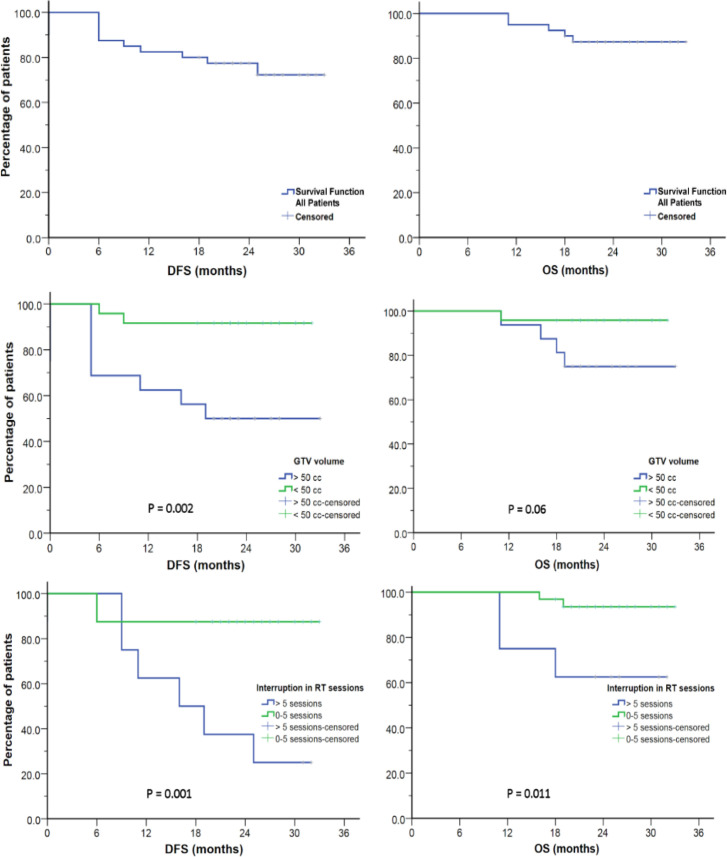
Kaplan Meier Survival Curves Showing Disease Free Survival (DFS) and Overall Survival (OS) for the Whole Group of Patients (Upper Row), According to GTV Size ( Middle Row) or According to Number of Interrupted Radiotherapy Sessions (Lower Row).

The 2 years DFS is estimated to be 77%. Analysis of the factors affecting DFS revealed that the only significant factors are; ECOG PS, interruption of RT sessions of > 5 sessions, GTV volume [p = 0.041, 0.002, and 0.001 respectively]. However in the multivariate analysis, none of these factors reached statistical significance. (Table 5 and Figure 2).

*Factors affecting overall survival*

The 2 years OS is estimated to be 87%. Analysis of the factors affecting OS revealed that the only factor that was statistically significant with a detrimental effect on OS is interruption of RT sessions for > 5 sessions [p = 0.011]. Large GTV volume (>50 cm3) was borderline significant [p = 0.06]. However on multivariate analysis, none of these factors reached statistical significance. (Table 5 and Figure 2).

**Table 1:** Patients and Tumoral Characteristics Included in Our Study

Variable / Factor	Number	Percent
Gender		
Male	26	65.0
Female	14	35.0
Age group		
< 60 years	34	85.0
> 60 years	6	15.0
Median age: 49 years. Range: 19 - 69 years		
Smoking history		
Yes	21	52.5
No	19	47.5
ECOG Performance Status		
0	17	42.5
1	21	52.5
2	2	5.0
ACE grading		
0	25	62.5
1	12	30.0
2	3	7.5
T-stage		
T1	4	10.0
T2	12	30.0
T3	16	40.0
T4	8	20.0
N-stage		
N0	3	7.5
N1	4	10.0
N2	26	65.0
N3	7	17.5
M-stage		
M0	40	100.0
Stage group (UICC 7th ed.)		
III	23	57.5
IVa	10	25.0
IVb	7	17.5
Site of primary lesion		
Nasopharynx	24	60.0
Oropharynx	7	17.5
Larynx	4	10.0
Hypopharynx	3	7.5
Paranasal Sinuses	2	5.0

**Table 2:** Analysis of Factors that Affect Locoregional Control (LRC)

Factor /Variable	Ratio of LRC	% of LRC	P-value *
Gender			
Male	22/26	84.6	0.471
Female	11/14	78.6	
Age group			
< 60 years	28/34	82.3	0.376
> 60 years	5/6	83.3	
Smoking history			
Yes	16/21	76.2	0.412
No	17/19	89.5	
ECOG PS			
0	16/17	94.1	0.163
1	16/21	76.2	
2	1/2	50.0	
ACE grade			
0	21/25	84.0	0.565
1	10/12	83.3	
2	2/3	66.7	
Stage			
III	20/23	87.0	0.326
IV	13/17	76.5	
Site of the primary			
Nasopharynx	18/24	75.0	0.135
Other	15/16	93.8	
Induction chemotherapy			
Yes	16/20	80.0	0.5
No	17/20	85.0	
Not receiving all concurrent chemotherapy cycles			
Yes	21/28	75.0	0.064
No	12/12	100.0	
Interruption in RT sessions			
0-5 sessions	29/32	90.6	0.02
> 5 sessions	4/8	50.0	
GTV volume			
< 50 cm^3^	23/24	95.8	0.001
> 50 cm^3^	10/16	62.5	

*Significance is calculated by 2-sided chi-square test.

**Table 3:** Early and Late Toxicities According to CTCAE v. 4.03

Factor	Highest grade	Number of patients	% of patients	Time point of highest grade toxicity*
Early toxicities				
Anemia	0	8	20.0	35% of cases occurred in week 4, and 30% in week 5 of RT
	1	24	60.0	
	2	8	20.0	
Neutropenia	0	8	20.0	30% of cases occurred in week 4, and 37.5% in week 5 of RT
	1	12	30.0	
	2	9	22.5	
	3	11	27.5	
Mucositis	2	10	25.0	40% of cases occurred in week 5, and 32.5% in week 6 of RT
	3	26	65.0	
	4	4	10.0	
Xerostomia	2	26	65.0	42.5% of cases occurred in week 6, and 47.5% in week 7 of RT
	3	14	35.0	
Pain	2	15	37.5	55% of cases occurred in week 5, and 25% in week 6 of RT
	3	25	62.5	
Laryngitis	1	3	7.5	27.5% of cases occurred in week 4, and 40% in week 5 of RT
	2	22	55.0	
	3	13	32.5	
Dysgeusia	2	40	100.0	50% of cases occurred in week 4, and 40% in week 5 of RT
Dysphagia	1	2	5.0	32.5% of cases occurred in week 4, and 57.5% in week 5 of RT
	2	19	47.5	
	3	19	47.5	
Thickening of salivary secretions	1	23	57.5	45% of cases occurred in week 5, and 27.5% in week 6 of RT
	2	15	37.5	
Radiation dermatitis	1	1	2.5	40% of cases occurred in week 5, and 52.5% in week 6 of RT
	2	33	82.5	
	3	6	15.0	
Late toxicities				
Tinnitus	1	16	40.0	
Neck edema	1	13	32.5	
	2	1	2.5	
Hoarseness	1	3	7.5	
	2	7	17.5	
Dental caries	1	4	10.0	
	2	2	5.0	
Xerostomia	1	20	50.0	
	2	16	40.0	
	3	2	5.0	
Dysphagia	1	26	65.0	
	2	10	25.0	
	3	2	5.0	
Trismus	1	5	12.5	
Dysgeusia	1	12	30.0	
	2	28	70.0	
Fibrosis	1	17	42.5	

*Applicable only for acute toxicities.

**Table 4:** Factors Affecting Development of Grade 3-4 Toxicities

Factor	Variable	Ratio of patients with high grade toxicities	% of patients with severe toxicity	*P*-value*
Gender	Male	20/26	76.9	0.507
	Female	12/14	85.7	
Age group	< 60 years	26/34	72.2	0.184
	> 60 years	6/6	100.0	
Smoking history	Yes	17/21	80.9	0.874
	No	15/19	78.9	
ACE grade	0	21/25	84.0	0.68
	1	9/12	75.0	
	2	2/3	66.7	
Induction chemotherapy	Yes	19/20	95.0	0.018
	No	13/20	65.0	
GTV volume	< 50 cm^3^	16/24	66.7	0.013
	> 50 cm^3^	16/16	100.0	

*Significance is calculated by 2-sided chi-square test.

**Table 5:** Analysis of Factors that Affect Disease Free Survival (DFS)& Overall Survival (OS)

Factor	Variable	2 years DFS	*P*-value*	Multivariate analysis	2 years OS	*P*-value*	Multivariate analysis
Age group	< 60 years	77	0.638		85	0.129	
	> 60 years	84			100		
ECOG PS	0	100	0.041	0.285	94	0.448	
	1	68			81		
	2	50			70		
ACE-27 grade	0	76	0.611		79	0.93	
	1	75			100		
	2	100			100		
Stage	III	79	0.607		96	0.076	
	IV	76			76		
Induction chemotherapy	Yes	72	0.14		100	0.129	
	No	85			82		
Not receiving all concurrent chemotherapy cycles	Yes	70	0.085		86	0.588	
	No	93			100		
Interruption in RT sessions	0-5 sessions	87	0.001	0.075	62.5	0.011	0.19
	> 5 sessions	38			93.5		
GTV volume	< 50 cm^3^	92	0.002	0.121	96	0.06	0.28
	> 50 cm^3^	50			75		

*Significance is calculated by Log rank test.

## Discussion

In developing countries, management of locally advanced head and neck squamous cell carcinoma (LA-HNSCC) represents a challenge for both patients and clinicians. The multiplicity of critical functions in this region greatly affects the patients’ quality of life and their adherence to the treatment protocol. One of the limitations of this study is the small sample size, and although the follow up period is short yet it is sufficient for evaluation of immediate locoregional control rates and side effects of this new approach in our region.

One of the important features of HNSCC is the ability of these tumors to grow rapidly which may compensate for radiation-induced cell loss through accelerated repopulation (Withers et al., 1988).So locoregional recurrence represents the predominant cause of failure in these tumors (Oksuz et al., 2011). Consequently, efforts should focus on developing a therapeutic approach aiming at improving locoregional control (LRC) for this group of patients.

Altered fractionation has been applied in an attempt to overcome this problem. Data from the RTOG 9003 trial enrolling 1076 patients and after 14 years of follow up, showed that hyperfractionation improved LRC and OS without increasing late toxicities compared to conventional fractionation (Beitler et al., 2014). The addition of chemotherapy to altered fractionation has also proven to be more efficacious than altered fractionation alone. A large meta-analysis including 32 trials with 10,225 patients showed an OS benefit of 12 months for the addition of concurrent chemotherapy to either conventional, accelerated or hyperfractionated RT (p < 0.0001) (Budach et al., 2006).

All these factors were taken in consideration in the design of this study in which concurrent chemoradiotherapy with simultaneous integrated boost (SIB) -as a sort of accelerated fractionation- was used for treatment of patients with LA-HNSCC. The study also reflects the epidemiological features of head and neck carcinomas in our region as the majority of patients were males (65%), with history of smoking (80%), no alcohol intake and nasopharynx being the commonest site (60%). This pattern is predominant in the South East Mediterranean and North African population (Ferlay et al., 2015).

One of the earliest experiences with IMRT was reported from University of California where 67 patients with nasopharyngeal carcinoma (NPC) were treated between the years 1995 and 2000. The 4-year estimates of LRC and OS were 98% and 88% respectively (Lee et al., 2001). LRC rates around 90% in NPC have been reported since then in other single-institutional studies; (Wolden et al., 2006; Tonoli et al., 2016) as well as in the multi-institutional RTOG 0225 study which used SIB, with 2.12 Gy per fraction delivered to the gross disease. After a median follow up of 2.6 years, the estimated 2-year locoregional DFS and OS rates were 89.3% and 80.2%, respectively (Lee et al., 2009). However all these series included patients with NPC only. Montejo et al, used IMRT with SIB and concurrent cisplatin or cetuximab to treat 43 patients with LA-HNSCC. With a median followup of 36.7 months, they reported 2-year LRC, DFS, and OS rates of only 73%, 61%, and 65% respectively (Montejo et al., 2011).

In our study, the 2-year LRC, DFS and OS were 82.5%, 77% and 87% respectively. With the majority of our patients having locally advanced NPC, our response and survival rates seem quite reasonable and acceptable. 

Studer et al., (2006) studied 115 patients with HNSCC treated by IMRT with SIB and concurrent weekly cisplatin 40mg/m2. They reported grade 3 mucositis in 15% of cases, grade 3 xerostomia in 10% and grade 3 dysphagia in 20% of cases. No early grade 4 toxicities were reported. 

In our series, high grade mucositis (grade 3 or 4) occurred in 75% of cases. Grade 3 xerostomia was observed in 35% and grade 3 dysphagia in 47.5% of cases. Our early side effects are quite high compared to the results of Studer et al., which is most probably related to the use of different assessment scales (WHO vs. CTCAE). In addition the former study was retrospective, with 30% of cases treated post-operatively and only 62% of cases had stage 3-4 diseases. 

Subgroup analysis of our patients showed that grade 3-4 mucositis was nearly universal in patients receiving induction chemotherapy (95% of cases), compared to 65% in patients not receiving induction therapy [p = 0.018]. Furthermore, a larger GTV (>50 cm3) resulted in significantly higher grade 3-4 acute toxicities [p = 0.013], therefore it is expected to have high incidence of grade 3-4 mucositis in patients with more advanced stages. While providing higher survival outcomes compared with conventional RT (Beadle et al., 2014), IMRT has resulted in significantly lower rates of late complications as shown from the results of the RTOG 0225 study where at 1 year, only 3% of patients had grade 3 xerostomia, and none had grade 4 xerostomia (Lee et al., 2009). A Swiss retrospective analysis reported high tolerance to SIB-IMRT with a remarkable 2% grade 3-4 late effects in elderly (80+) patients (Brown et al., 2016).

In our series, grade 3-4 late radiation toxicities were noted in 10% of cases. Only 5% of cases had grade 3 xersotomia and 5% had grade 3 dysphagia after 6 months from the end of RT. No grade 4 toxicities were reported. 

The enthusiasm towards induction chemotherapy in LA-HNSCC is fading nowadays, since it has failed to provide any survival benefit, while significantly increasing the acute toxicities (Zhang et al., 2015; Takacsi-Nagy et al., 2015). In the MACH-NC meta-analysis, concurrent chemotherapy has provided 6.5% absolute survival benefit (HR 0.81; 95% CI 0.78-0.86; p = 0.001) compared to induction regimen (Pignon et al., 2009).

Our results revealed that failure to adhere to the planned protocol was significantly higher in patients who received induction chemotherapy due to the significantly higher acute toxicities; either hematological or non-hematological, [p = 0.001 and 0.058 respectively]. We found that 90% of patients who received induction chemotherapy have omitted one or more cycles of the concurrent chemotherapy and 75% of these patients had interruption in their radiotherapy schedule, with statistically significant differences as compared with patients who didn’t receive induction therapy [p = 0.006 and 0.001 respectively]. However this didn’t significantly affect LRC or survival, (see Tables 2 and 5). Longer follow may be required to assess the effect of induction chemotherapy on LRC, DFS and OS.

The effect of GTV volume on tumor control was described in various studies, for example in the study of Studer et al, the mean GTV volume in patients with local failure was 63 cm^3^ compared to 32 cm^3^ in locally controlled patients [p < 0.01] (Studer et al., 2006). 

This effect was more obvious in our study, where patients with GTV volume >50 cm3 were found to have significantly lower local control rate and 2 years DFS compared to patients with GTV volume <50 cm3. These patients seem to have lower 2 years OS also.

Medical records of 3,864 patients with HNSCC were studied by Fesinmeyer et al., (2010) to evaluate the influence of RT interruption on survival. They concluded that interruption may influence the survival time among patients completing a full course of RT. A more recent analysis prolonged radiotherapy treatment time was associated with a worse OS (HR 1.25; 95% CI 1.14-1.37; P<0.001) while accelerated radiotherapy was associated with an improved OS (HR 0.84; 95% CI 0.73-0.97; P=0.02) relative to standard treatment times (Shaikh et al., 2016).

In our study, long radiation interruptions were associated with significantly lower LRC rate, lower 2 years DFS and lower 2 years OS compared to patients without interruption, confirming the detrimental effect of radiation interruptions in patients with LA-HNSCC.

In conclusion, the use of SIB-IMRT as accelerated RT schedule with weekly cisplatin seems tolerable and results in good control rates. Large tumors (>50 cm^3^) and long radiation interruptions (> 5 sessions) are associated with significantly lower locoregional control, DFS and OS.

Induction chemotherapy seems to have a negative impact on patients’ compliance and tolerance to concurrent chemoradiotherapy approach, with a possible indirect effect on DFS and OS through increasing treatment interruptions. Therefore its use should be limited to clinical trials. 

In a developing country, combating financial, social and infrastructure related factors that leads to delay in initiating treatment and prolonged treatment interruptions has to be the focus of any health care plan aiming to succeed in the fight against head and neck cancers.
